# Triazine-Based Conjugated Microporous Polymers With Different Linkage Units for Visible Light–Driven Hydrogen Evolution

**DOI:** 10.3389/fchem.2022.854018

**Published:** 2022-03-25

**Authors:** Qiannan Sheng, Xiujuan Zhong, Qianqian Shang, YunYun Dong, Jinsheng Zhao, Yuchang Du, Yu Xie

**Affiliations:** ^1^ College of Chemistry and Chemical Engineering, Liaocheng University, Liaocheng, China; ^2^ Key Laboratory of Jiangxi University for Applied Chemistry and Chemical Biology, College of Chemistry and Bioengineering, Yichun University, Yichun, China; ^3^ College of Environment and Chemical Engineering, Nanchang Hangkong University, Nanchang, China

**Keywords:** conjugated microporous polymers, triazine, photocatalytic, hydrogen production, linkage unit

## Abstract

Conjugated microporous polymers (CMPs), as a kind of two-dimensional material, have attracted extensive attention due to their advantages in visible light–driven photocatalytic splitting of water for hydrogen evolution. However, improving the microstructure and electronic structure of the material to enhance their photocatalytic performance for hydrogen evolution remains a challenge. We designed and reported two analogous CMPs including CMP-1 and CMP-2 that contain triazine and dibenzothiophene-*S*,*S*-dioxide units, which were prepared by Pd-catalyzed Suzuki-Miyaura coupling reaction. The main difference of two CMPs is that the triazine units are connected to benzene unit (CMP-1) or thiophene unit (CMP-2). Both of the CMPs exhibit excellent light capture capability, and compared with CMP-2, CMP-1 has faster separation rates and lower recombination rates for the charge carriers (electron/hole), and then, a higher hydrogen evolution rate was obtained from water decomposition reaction. We find the H_2_ production rate of CMP-1 can be up to 9,698.53 μmol g^−1^h ^−1^, which is about twice of that of CMP-2. This work suggests that molecular design is a potent method to optimize the photocatalytic performance toward hydrogen evolution of the CMPs.

## Introduction

Hydrogen is a form of clean and high density (120 MJ/kg) energy. If produced efficiently from sunlight-driven photocatalytic splitting of water, then it is anticipated to play an important role in the global effort of realizing carbon neutralization (Fujishima and Honda, 1972; Chen et al., 2010; Linares et al., 2014). In the photocatalytic process, semiconductor catalysts are required to absorb photons and result in the separation and transfer of charge carriers, and the photogenerated electrons are transferred to the protons to produce hydrogen (Turner et al., 2008; Wang et al., 2010; Dincer and Acar, 2015; Shi et al., 2017; Wang L. et al., 2018). Since Fujishima and Honda (1972) found that the decomposition of water into hydrogen and oxygen occurs under the irradiation of light on TiO_2_, inorganic semiconductors such as metal oxides and sulfides have been investigated mostly for their photocatalytic ability in water splitting (Chen et al., 2017; Yu et al., 2018; Tsang et al., 2019). However, inorganic catalysts, such as TiO_2_ or CdS, are poor light absorbers, susceptible to light corrosion, and often environmentally unfriendly. These limitations have seriously hindered their practical applications and further development (Kudo and Miseki, 2009; Osterloh, 2013; Wang et al., 2019). In recent years, organic polymer catalysts represented by graphitic carbon nitride (GCN) have been well developed, which have the advantages of adjustable energy level and spectral absorption range, environmentally friendly, and the wide availability of raw materials (Dai and Liu, 2020; Jayachandran and Chou, 2020). At present, the pure GCN developed are not competent with the commercial requirements of photocatalytic hydrogen production due to their limited sunlight absorption, high recombination rate of electron/holes, and low photocatalytic activities (Wang et al., 2009; Lin et al., 2016; Cui et al., 2020). Some other types of polymer-based catalysts are also developed over the same period, which are designed and synthesized based on many considerations including the specific active sites (or units), stereo configurations, porous structures, and hydrophilicity (Han et al., 2015; Huang et al., 2015; Yu et al., 2017).

In recent years, certain types of conjugated polymers, such as GCN, conjugated microporous polymers (CMPs) (Wang Z. et al., 2018; Xu et al., 2018; Xiao et al., 2020), linear conjugated porous polymers (CPPs) (Sprick et al., 2018; Bai et al., 2019; Ting et al., 2019), and covalent triazine frameworks (CTFs) (Guo et al., 2019; Huang et al., 2019), have been developed for H_2_ evolution. These polymers are adjustable in energy level and spectral absorption range, are environmentally friendly, and can be produced from the widely available raw materials (Dai and Liu, 2020; Jayachandran and Chou, 2020). They can be designed and synthesized to feature specific active sites (or units), stereo configurations, porous structures, hydrophilicity, and dispersibility (Han et al., 2015; Huang et al., 2015; Yu et al., 2017).

**SCHEME 1:**
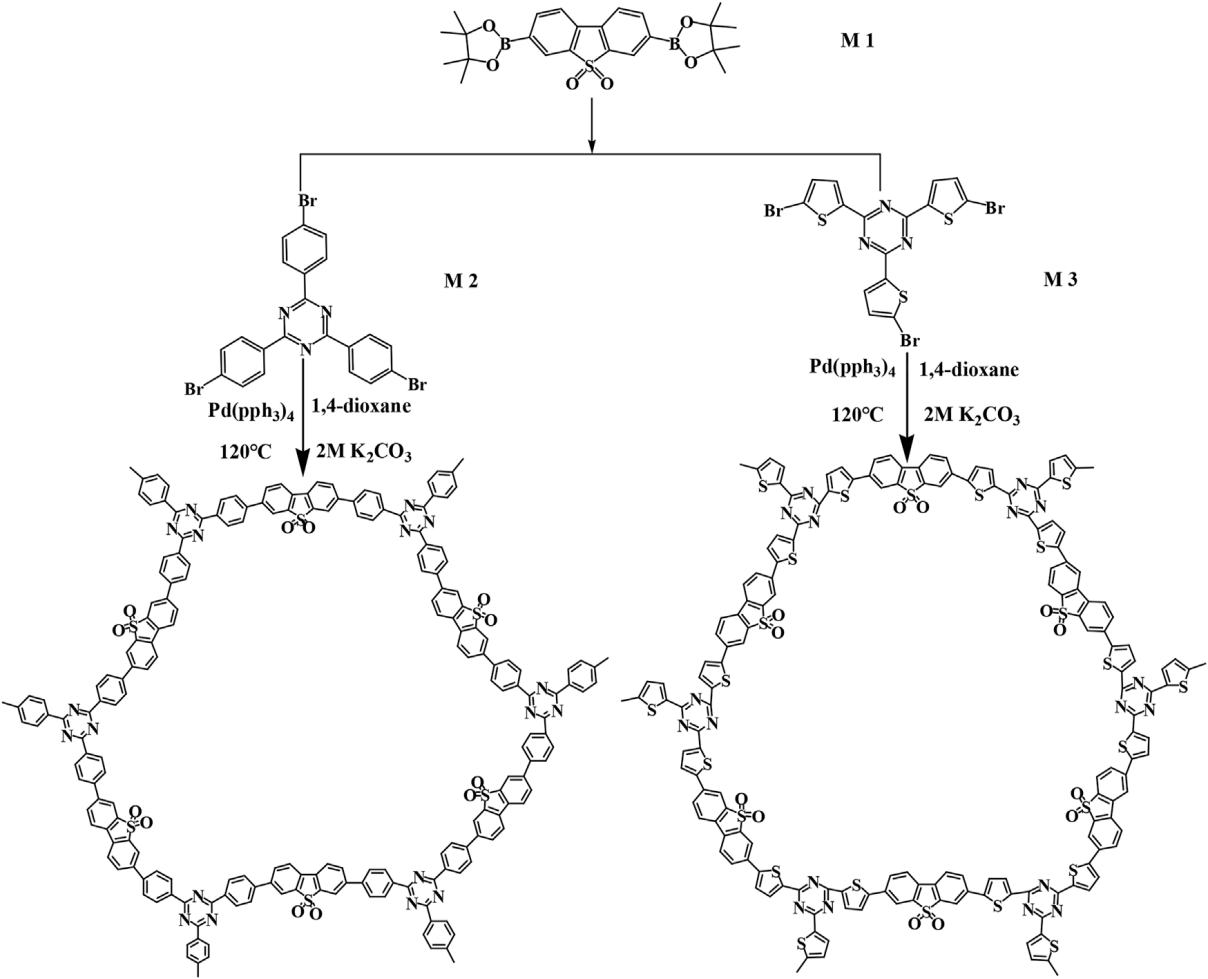
Synthesis of CMP-1 and CMP-2.

Dibenzothiophene-*S*,*S*-dioxide (FSO) has been frequently used as the building unit for the construction of highly active photocatalysts for hydrogen evolution from water splitting because of its aromatic structure, electron deficient character, and its unique function as electron output tentacle (Dai et al., 2018; Zhao et al., 2018). The introduction of the FSO units can help align the energy level for hydrogen evolution and enhance the efficiencies for the separation and transportation of excitons. So far, researchers have synthesized a variety of conjugated polymers containing FSO units, many of which exhibit impressive photocatalytic activities toward hydrogen evolution. Chen et al. found that the CPP P-FSO (Supplementary Scheme S1) gives a hydrogen evolution rate (HER) of 8,000 μmol g^−1^ h^−1^ (Lan et al., 2019). Wang et al. reported that the homopolymer of FSO can give a high HER up to 44.2 mmol h^−1^ g^−1^ under visible light irradiation (Supplementary Scheme S1) (Shu et al., 2020a). A CMP material S-CMP3 exhibited a HER of 6076 μmol g^−1^ h^−1^ under visible light irradiation (Supplementary Scheme S1) (Sprick et al., 2019). Another similar CMP PyDF was also reported, which showed an attractive HER of 18.93 mmol h^−1^ g^−1^ under visible light (Supplementary Scheme S1) (Gao et al., 2020). The conjugated polymers containing 1,3,5-triazine unit have been frequently used as photocatalysts, and the nitrogen atoms in the triazine unit have been considered as the active site for the HER reaction because they have lone pair electrons, electron deficient, and hydrophilicity character. Tan et al. prepared some covalent triazine containing frameworks with a D-A_1_-A_2_ configuration and the highest HER up to 19.3 mmol g^−1^ h^−1^ (Guo et al., 2019). From the study of Jin et al., the polymer CTF-HUST-C1 (Scheme 1), a crystalline covalent framework containing triazine unit, gives a HER of 5,100 μmol g^−1^ h^−1^ (Wang et al., 2017).

Therefore, in this work, we synthesized two CMPs, which are constructed from two different triazine derivatives as building units and FSO as linking units, and analyzed their structure and photocatalytic performances. Using triethanolamine (TEOA) as the sacrificial agent and Pt as the co-catalyst, the results showed that CMP-1, a CMP made from benzene rings connecting to a triazine unit, has a HER of 9,699 µmol g^−1^h^−1^, which is higher than that of CMP-2, an analogous polymer with thiophene unit as the linkage unit.

## Materials and Methods

### Instruments and Reagents

The analytical methods and instruments can be seen in the Supporting Information (SI). The reagents and their available sources, and the synthetic procedures for M1 and M3 are also given in SI part.

### Synthesis of CMP-1

A 100-ml round bottom flask was charged with 300 mg of M1 (0.6408 mmol), 233.3 mg of M2 (0.4272 mmol), 4 ml of K_2_CO_3_ (2 M) solution, 16 ml of dioxane, and 37 mg of Pd (pph_3_)_4_ catalyst (5% mol/mol of M1) (Scheme 1) (Zhao et al., 2018). The above solution was degassed three times to replace the atmosphere in the flask with nitrogen gas. The flask was heated to reflux in oil bath and be stirred continuously for 48 h. The raw product was cooled down to room temperature and was recovered by filtration, and then, it was washed with ethanol and distilled water successfully and then dried at 100°C under vacuum for 24 h. The crude product was purified by Soxhlet extraction for 24 h, and the solvent used is chloroform, after which the purified product was dried at 80°C overnight. Finally, 229 mg of yellow powder was obtained as CMP-1 with a yield of 78% (Wang Z. et al., 2018).

### Synthesis of CMP-2

First, 241 mg of M3 and 300 mg of M1 were added into a round-bottom flask, and then, 4 ml of K_2_CO_3_ (2 M) solution, 16 ml of dioxane, and 0.037 g of Pd (pph_3_)_4_ were also added subsequently (Scheme 1). Second, the mixture was degassed for three times and kept in nitrogen gas environment with the help of a balloon, and the flask was then immersed in an oil bath and was refluxed with magnetic stirring for 48 h (Shu et al., 2020a). After that, the material was obtained by filtration to get a yellow color powder. Last, the sample was dried at 80°C under vacuum for 24 h to get the raw product. The crude product was washed for 24 h with chloroform in a Soxhlet extractor (Shu et al., 2021).

### Photocatalytic Hydrogen Production

All the photocatalytic experiments were carried out in a glass-closed gas circulation system with a 100-ml Pyrex glass reaction vessel as the reactor, and the temperature was maintained at 10°C by a cyclic condensation device. To the above reaction vessel, 20 mg of photocatalyst, 10 ml of TEOA (as sacrificial agent), 10 ml of N-methylpyrrolidone (NMP), and 40 ml of distilled water were added. The catalyst was completely dispersed by ultrasound for 30 min, 24 µl of chloroplatinic acid was added, and then the stirred solution was irradiated with a 300-W Xe lamp (CEL-HXF300) for 3 h to load Pt nanoparticles (3%, w/w) on the catalyst. The Pt-modified photocatalyst can be recovered by filtration and can be used in the following photocatalytic hydrogen evolution experiments. The Xe lamp was equipped with a 420-nm cutoff filter (CEL-UVIRCUT420) to evaluate the visible light–induced photocatalytic activity of the catalysts. Before the light irradiation, the reaction system was pumped for at least 20 min, as far as possible to get rid of the dissolved oxygen in solution, and kept it in a vacuum state. Then, the suspension was stirred for 3 h and irradiated, and the hydrogen evolution was sampled and analyzed every 30 min. The hydrogen produced was detected by gas chromatograph (GC-7920) with a TCD detector. The photocatalysis stability was carried out for five periods with 3 h for one period.

## Results and Discussion

### Morphological and Structure Characterization

**FIGURE 1:**
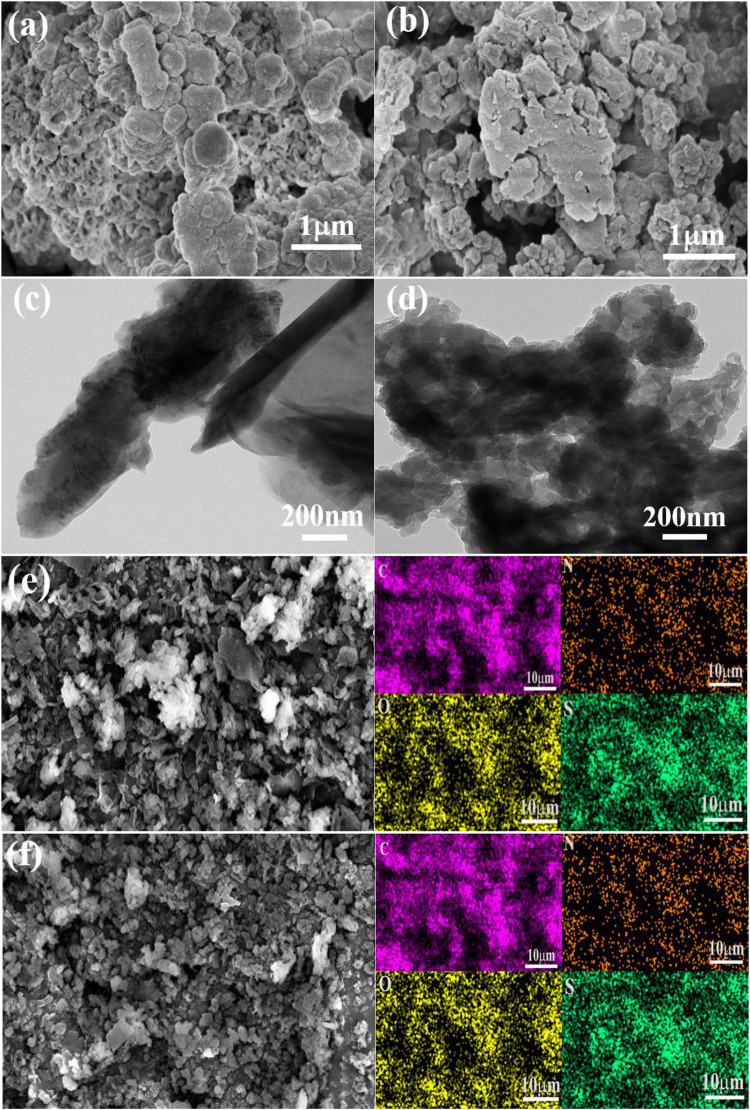
SEM image for CMP-1 **(A)** and CMP-2 **(B)**. TEM image of CMP-1 **(C) and** CMP-2 **(D)**. EDS element mapping images of CMP-1 **(E)** and CMP-2 **(F)**.

The microstructures of the CMPs are characterized by scanning electron microscope (SEM) and transmission electron microscope (TEM). According to SEM (Figures 1A,B) and TEM (Figures 1C,D), the two CMPs have similar morphology and the small particles of them stacked together to form the irregularly shaped aggregation. In addition, the element mapping images (Figures 1E,F) are given under SEM by energy-dispersive X-ray spectroscopy (EDS), and it can be seen that four elements C, N, O, and S are evenly distributed in the CMPs. The percentage contents of the four elements are shown in Supplementary Figure S3.

**FIGURE 2:**
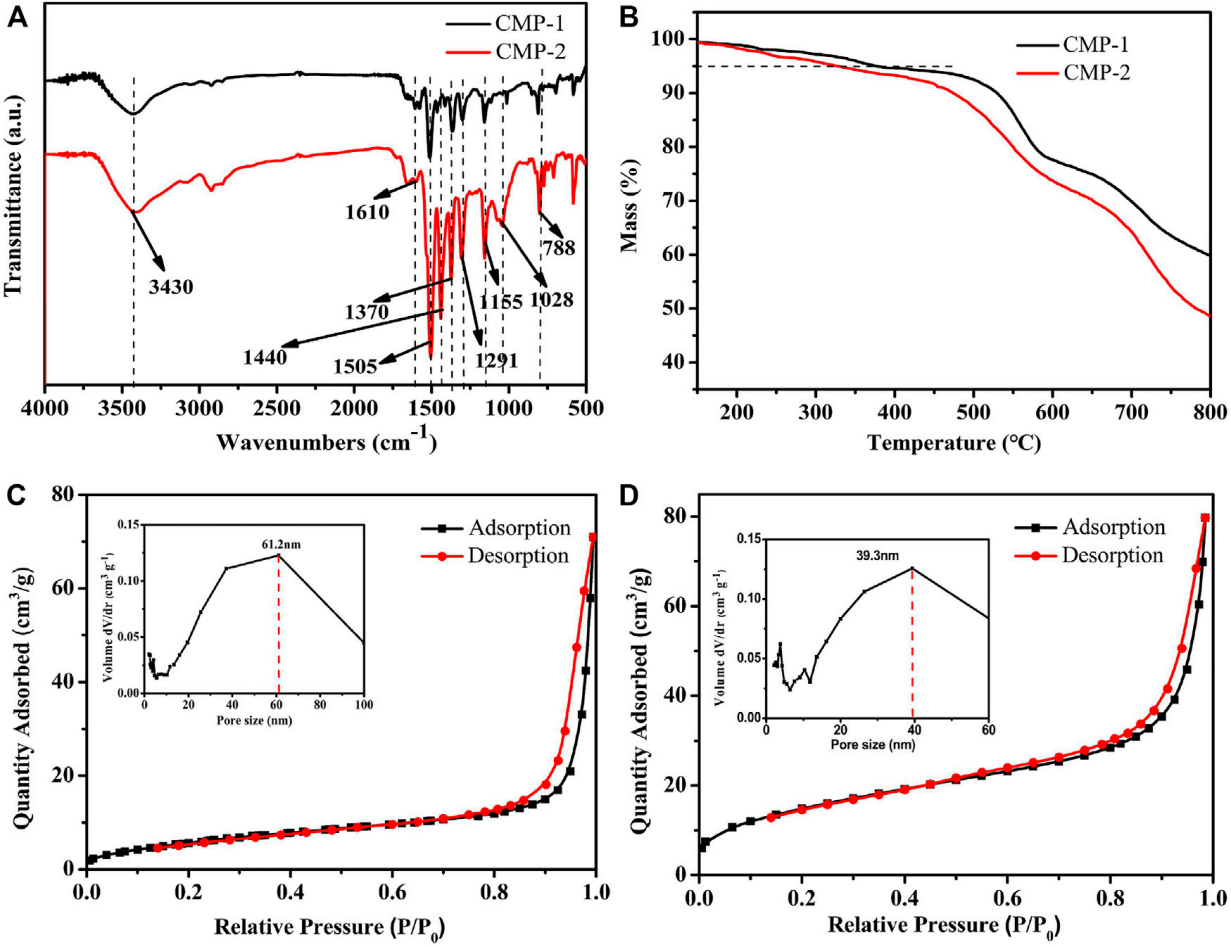
**(A)** FT-IR spectra of CMPs. **(B)** TGA curves of CMPs. Nitrogen adsorption–desorption isotherms at 77 K and pore size distributions (insets) of **(C)** CMP-1 and **(D)** CMP-2.

Fourier Transform infrared (FT-IR) was used to support the successful preparation of the two CMPs. As shown in Figure 2A, a wide peak around 3,430 cm^−1^ is observed, which could be attributed to the characteristic O-H stretching vibration from the trace moisture adsorbed by the CMPs. There are two transmission peaks at 1,505 and 1,370 cm^−1^, which belong to the stretching vibration of C-N and the in-plane stretching vibrations of the triazine ring, respectively (Guo et al., 2019; Gao et al., 2020). The strong peaks at 1,291 and 1,155 cm^−1^ correspond to the characteristic stretching peak of O=S=O group of the FSO unit (Zhang et al., 2020; Shu et al., 2021). The peak at 1,610 cm^−1^ is attributed to skeleton vibrations of aromatic rings. The peak at 788 cm^−1^ is the out-of-plane bending vibration of the C-H bonds, and the peak at 1,028 cm^−1^ is attributed to in-plane bending vibrations of the C-H bonds. The above peaks are the characteristic transmittance peaks shared by the two CMPs, which is also consistent with their similar structures. Different from CMP-1, the CMP-2 exhibits a strong peak at 1,440 cm^−1^, which belongs to the skeletal vibration of the thiophene unit in CMP-2 (Zhang et al., 2020). From the above analysis, it can be confirmed that the two CMPs are successfully obtained. Supplementary Figure S4 gives the FT-IR pictures of the CMPs after the photocatalytic reaction, which is identical to that of the as prepared CMPs. The PXRD pattern (Supplementary Figure S5) shows that all peaks of the as prepared polymers are broad, indicating the amorphous nature of the materials.

Thermogravimetric analysis (TGA) (Figure 2B) was used to analyze the thermal properties of the as prepared CMPs. Because of the rigidity of the polymer skeleton, both of the two CMPs have high initial decomposition temperatures (T_id_), i.e., 381°C for CMP-1 and 328°C for CMP-2. It is obvious that the T_id_ of CMP-1 is slightly higher than that of CMP-2, although only the benzene unit in CMP-1 was replaced by the thiophene unit in CMP-2. In addition, the characteristics on the surface porosity of the CMPs are also studied. Nitrogen adsorption–desorption experiments were carried out at 77 K; the two polymers showed a similar shape for their isotherms; as shown in Figures 2C,D, the two curves are consistent with the type III isotherm. At the low relative pressure region, there is a small amount of absorption, indicating the existence of some micropores. In addition, at the higher P/P0 region (0.85–1.0), the isotherm rises rapidly (likely due to capillary condensation of N_2_) and hysteresis was observed for the desorption of N_2_. The hysteresis in the desorption curve is due to the elastic deformation or swelling behavior caused by the N_2_ adsorption process (Zhou et al., 2019). The Brunner-Emmet-Teller (BET) surface areas of CMP-1 and CMP-2 are 54.77 and 22.92 m^2^/g, respectively. In the embedded diagram of Figures 2C,D, it can be seen that the pore size distribution of CMP-1 is mainly between 10 and 100 nm and is centered at 61.2 nm, and pore size distribution for CMP-2 is mainly between 10 and 70 nm and is centered at 39.3 nm. In addition, there are few micropores within the CMPs, as shown by the minor peaks at less than 10 nm (Zhang et al., 2020).

### X-Ray Photoelectron Spectroscopy (XPS) Analysis

**FIGURE 3:**
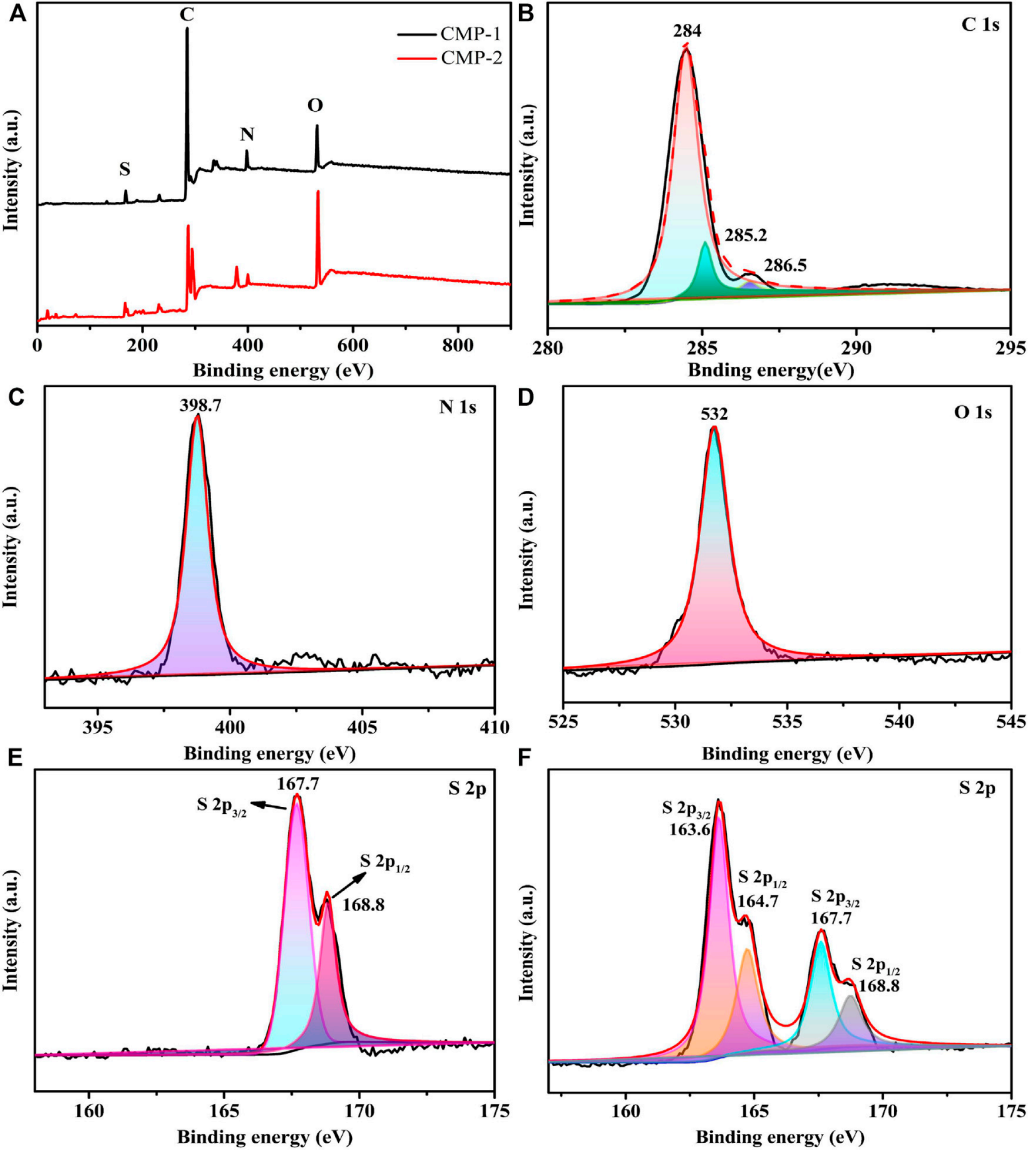
XPS survey spectrum **(A)**; high resolution XPS spectra of C 1s region **(B)**, N 1s region **(C)**, O 1s region **(D)**, and S 2p region of CMP-1 **(E)**; and high resolution XPS spectra of S 2p region of CMP-2 **(F)**.

XPS is used to analyze the surface compositions and element states of two CMPs. It can be seen from the survey spectrum (Figure 3A), both of two CMPs contain the elements of C, N, O, and S, which is consistent with the above EDS data. Figures 3B–E correspond to the XPS spectra of C 1s, N 1s, O 1s, and S 2p, respectively, for CMP-1. After the peak differentiating analysis, three definite peaks can be distinguished from the C 1s spectrum of CMP-1 (Figure 3B), and the binding energies (BEs) are 284.0, 285.2, and 286.5 eV, which belong to the carbon atoms in the C-C/C=C, C=N, and C-S, respectively. For the N 1s spectrum (Figure 3C), the peak with a BE value of 398.7 eV belongs to the pyridine nitrogen atom (-C=N-C) in the triazine ring. As shown in Figure 3D, the peak with a BE value of 532 eV was identified, which belong to the O 1s of the oxygen atom in the O=S unit within CMP-1. Two distinct peaks are identified in the S 2p spectrum (Figure 3E), and their BE values are 167.7 and 168.8 eV, respectively, which correspond to S 2p_3/2_ and S 2p_1/2_ of the sulfur atom in the O=S=O unit in the CMP-1. The above elements and their corresponding valence states confirmed the formation of CMP-1. The survey scan and the high resolution XPS spectra of the C, N, and O elements of CMP-2 are identical to that of CMP-1, which are consistent with their structural differences, lying in the branched unit, i.e., benzene for CMP-1 and thiophene for CMP-2. To identify the structure of CMP-2, the S 2p spectrum is given in Figure 3F; the former two peaks with lower BE values at 163.6 and 164.7 eV correspond to the S 2p_3/2_ and the S 2p_1/2_ of the sulfur atom in the C-S-C bond of the thiophene unit and the two latter peaks with higher BE values are found at 167.7 and 168.8 eV, respectively, which correspond to the S 2p_3/2_ and the S 2p_1/2_ of the sulfur atom in the O=S=O unit. The existence of two valence sulfur atoms is completely consistent with the structure of CMP-2, which confirms the successful preparation of CMP-2 (Shu et al., 2020b).

### Band Structure Analysis

**FIGURE 4:**
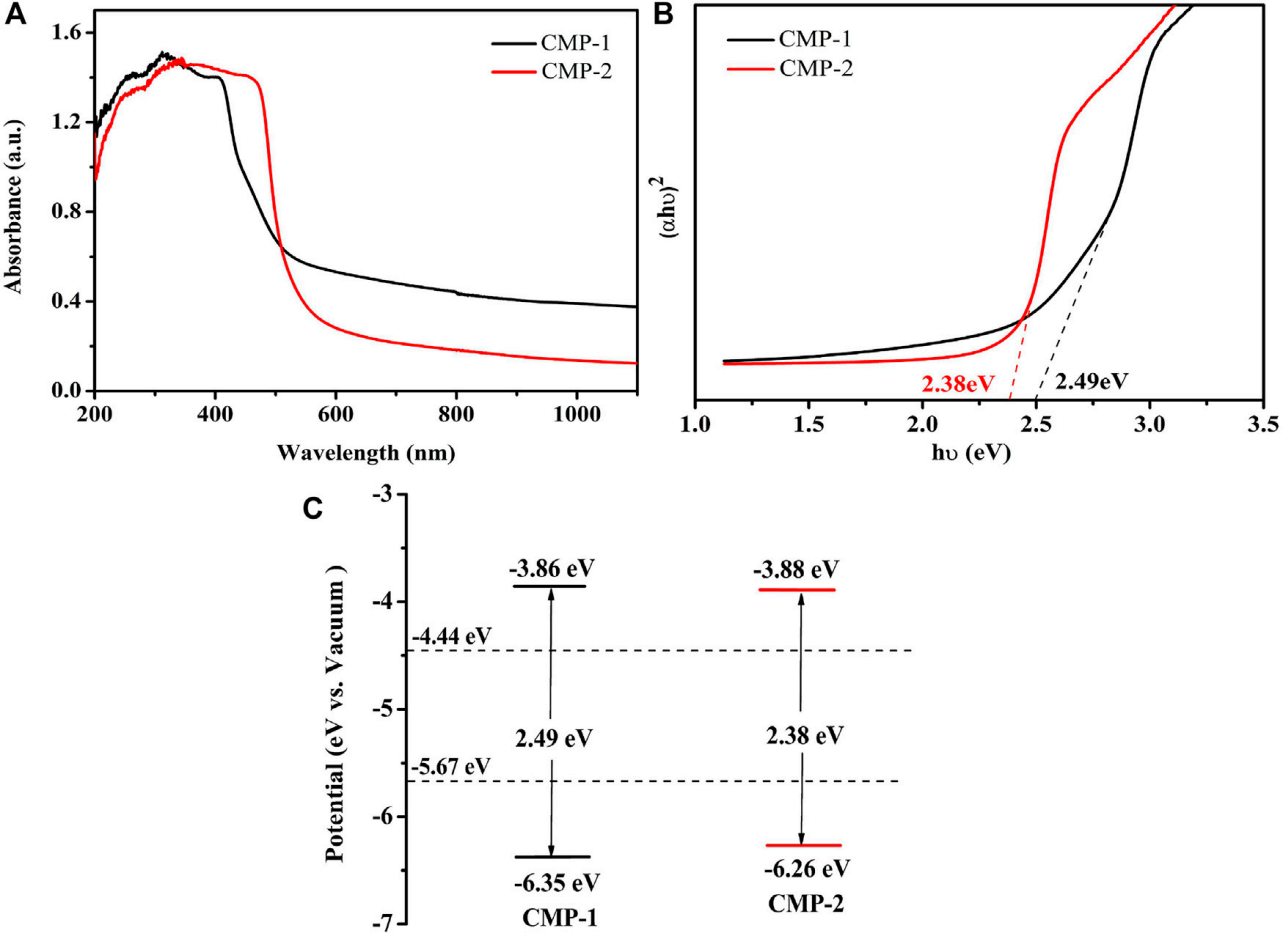
**(A)** UV-vis absorption spectra of CMP-1 and CMP-2. **(B)** (*αhv*)^2^ vs. *hv* curve of CMP-1 and CMP-2. **(C)** The band structure diagram of the CMPs.

For further explore the catalytic performance of the prepared CMPs, the absorbances of them were analyzed by UV-Vis diffuse reflectance spectra (UV-Vis-DRS) (Figure 4A). The two CMPs have a wide absorption range, the coverage for CMP-1 is 300–599 nm, which, for CMP-2, is 300–560 nm; both have a strong absorption in the visible range. The following Kubelka–Munk formula was used to draw the Tauc plot (Cui et al., 2020):

$$ahv=A{\left(hv-E_{g}\right)}^{2}$$ (1)

From the Tauc plot as shown in Figure 4B, the optical band gaps of the polymers are calculated to be 2.49 and 2.38 eV, respectively, for CMP-1 and CMP-2. The CMPs were also studied by cyclic voltammetry (Supplementary Figure S6), the onset reduction potentials (*E*_onset,re_) of them were determined, and the lowest unoccupied molecular orbital (LUMO) levels were estimated to draw their band structure diagrams. In addition, the relevant calculations were done using the following formula (Zhang et al., 2020):

$$E_{LUMO}=-e\left[4.8+\left(E_{onset,re}-0.5\right)\right]$$ (2)

$$E_{HOMO}=E_{LUMO}+E_{g}$$ (3)

In Eq. 3, *E*_g_ refers to the optical band gaps of the CMPs, the value of 0.5 V is the half wave potential of ferrocene [*E* (Fc/Fc^+^) vs. Ag/AgCl], and the *E*_onset,re_ values of CMP-1 and CMP-2 are −0.44 and −0.42 V (vs. Ag/AgCl), respectively. The LUMO energy levels of the CMPs are calculated to be −3.86 and −3.88 eV (vs. vacuum), respectively, and the highest occupied molecular orbital (HOMO) levels of two CMPs were calculated to be −6.35 and −6.26 eV, respectively, for COF-1 and COF-2. Then, the band structure diagrams of the polymers are calculated and drawn, as shown in Figure 4C. The Mott–Schottky curve of the two CMPs was tested at different frequencies (Supplementary Figure S7), both of the materials have a positive slope in the graph regardless of the changes, which is the characteristic of the n-type semiconductors, and the flat band potential (V_fb_) of CMP-1 and CMP-2 measured from the M-S plots were −0.59, −0.57 V vs. Ag/AgCl, corresponding to −0.38, −0.36 V vs. NHE, respectively, which is equal to the Fermi level (EF) for an n-type semiconductor (Shu et al., 2020b). Meanwhile, the conduction bands (CBs) of the polymers CMP-1 and CMP-2 are calculated to be −0.58 eV (−3.86 eV vs. vacuum) and −0.56 eV (−3.88 eV vs. vacuum), respectively. Combined with the band gap value, the valence bands (VBs) of CMP-1 and CMP-2 are calculated to be 1.91 eV (−6.35 eV vs. vacuum) and 1.82 eV (−6.26 eV vs. vacuum), respectively. The HOMO and LUMO data from the Mott–Schottky are consistent with the data from the cyclic voltammetry (CV) results.

**FIGURE 5:**
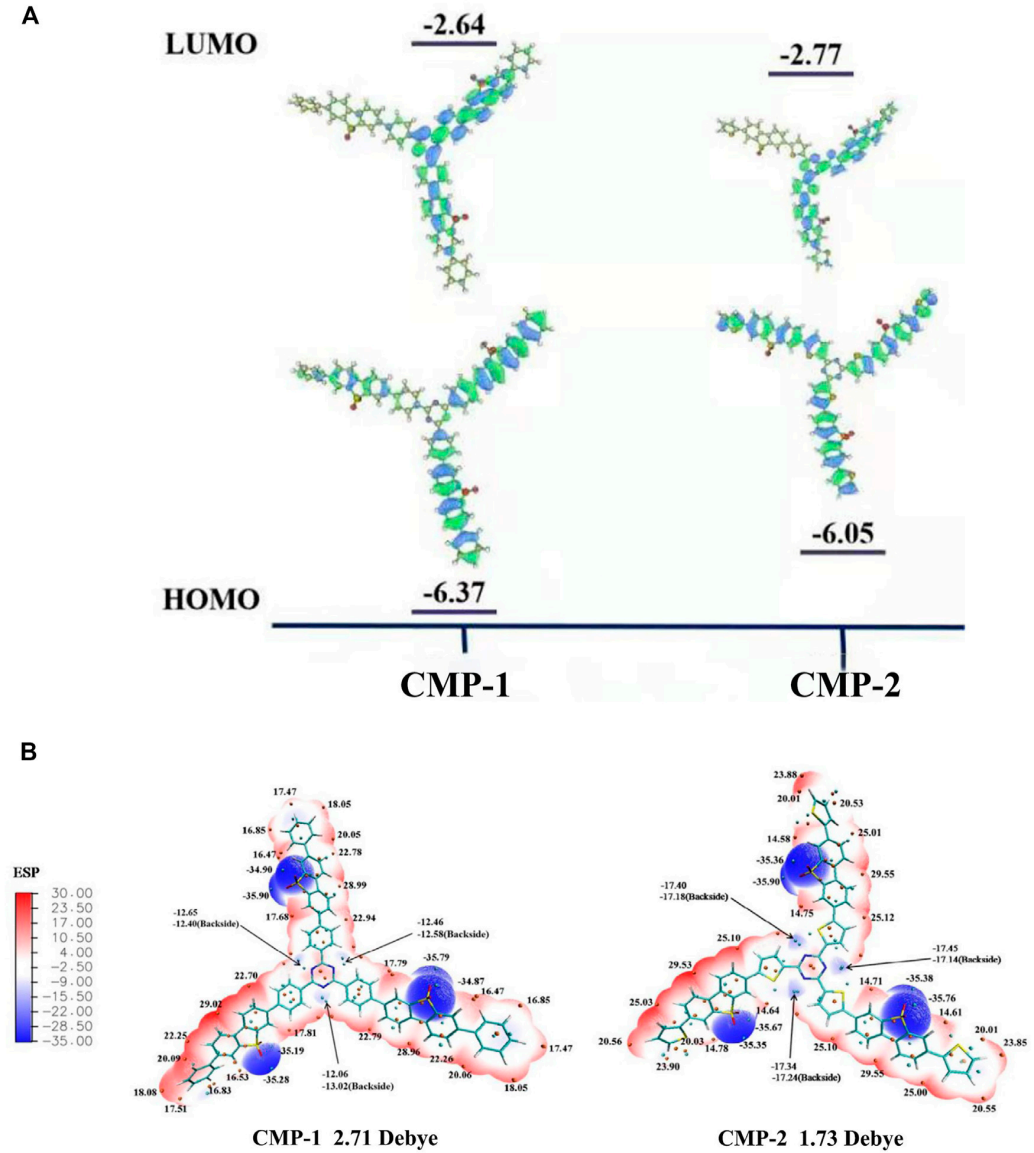
**(A)** HOMO and LUMO orbits distribution of the polymers from DFT calculation. **(B)** Diagram of molecular dipoles and surface electrostatic potential in CMP-1 and CMP-2.

Theoretical calculation (density functional theory (DFT)) was conducted on the basic repeated unit of two CMPs to get useful information on their electronic energy levels. For the fragmental unit of CMP-1, the electron density of its HOMO orbital is mainly delocalized over the benzene-FSO-benzene moiety. For the LUMO orbital of CMP-1, its electron density mainly resides on the π* orbital of the triazine-benzene-FSO moiety (Figure 5A). CMP-2 has a similar electronic structure as that of CMP-1 (Figure 5A). CMP-1 has a slightly higher CB and a lower VB than that CMP-2 (Figure 5A), which support the experimental studies of two CMPs. According to the band gap structure discussed above, CMP-1 has both the higher reduction ability and oxidation ability than that of the CMP-2, although the former has a slight narrow light absorption range than the latter one. The intramolecular dipoles of two CMPs could be calculated from the surface electrostatic potentials of them (Figure 5B), because the CMPs contained both electron donor units (benzene or thiophene) and electron acceptor units (triazine and FSO unit), which contributed to the formation of internal electric field. As calculated, molecular dipoles are 2.71 and 1.73 debye, respectively, for CMP-1 and CMP-2. The increase in molecular dipoles has a positive effect on the separation and transfer of photogenerated carriers, which would result in the higher HER of CMP-1 and CMP-2. The change of the constituent units in the molecular structure would regulate the electronic structures and photocatalytic activities of the catalysts (Ye et al., 2020).

### Photo‐Induced Charge Carriers and Their Transportation

**FIGURE 6:**
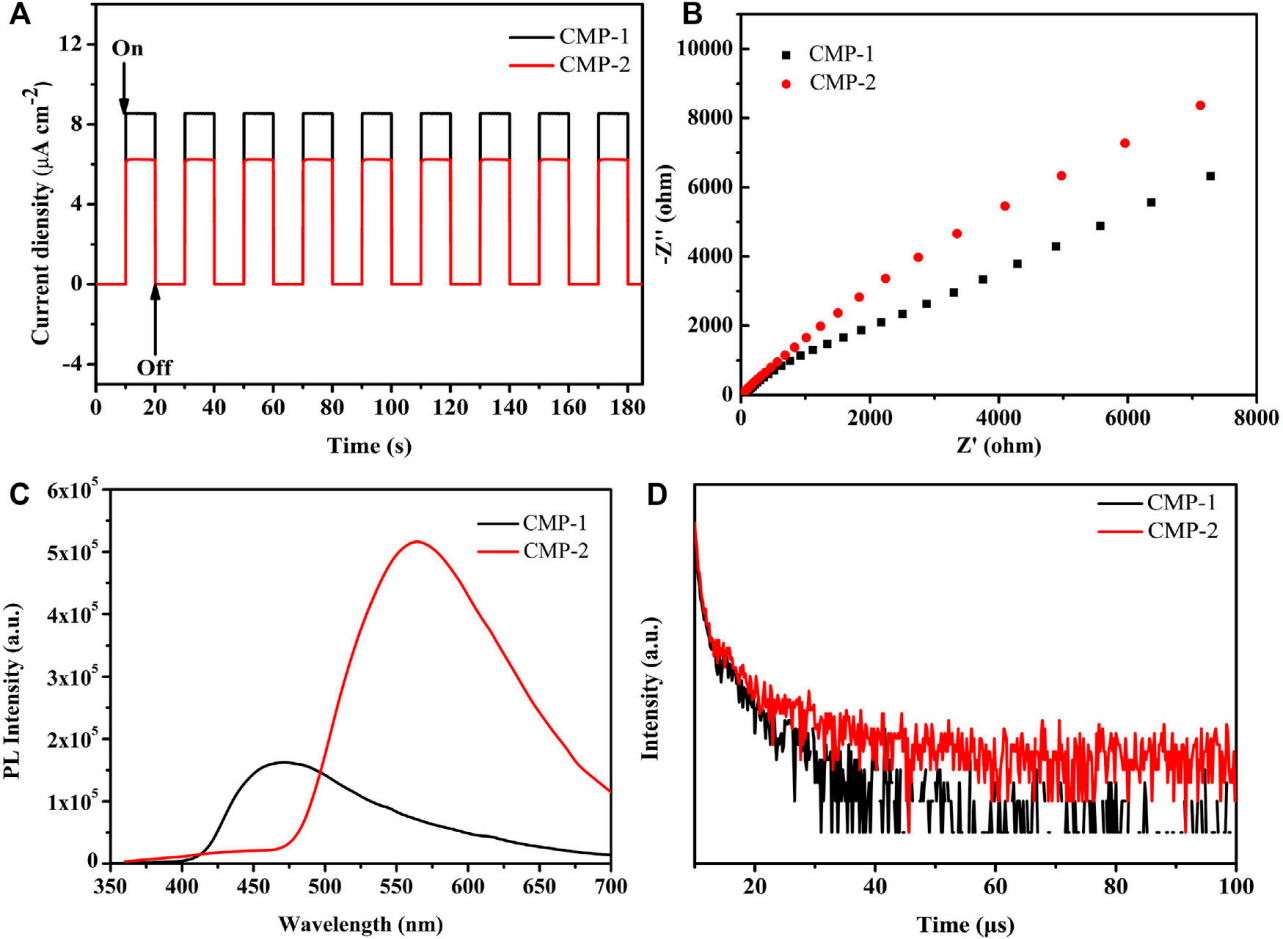
**(A)** Photocurrent of the CMPs (in 0.5 M Na_2_SO_4_); **(B)** EIS Nyquist plots of the CMPs; **(C)** PL spectra of the CMPs; **(D)** time-resolved PL spectra of the CMPs.

The photoelectron separation efficiencies of the polymers are also studied. Figure 6A shows the transient photocurrents of the two polymers, which were induced by a lamp source equipped with a 420-nm filter and with a biased voltage of 0 V on the photocatalyst-coated ITO electrode. The illumination time and the interval time are 10 s, respectively, and the amplitudes of the photocurrents for both catalysts remained stable levels up to nine cycles of the on-off illumination switch, which indicates that the photocatalysts have stable efficiencies for the separation and generation of the photogenerated carriers (Huang et al., 2015).

Besides, it is clearly shown that CMP-1 has a higher photocurrent response than that of CMP-2 (Figure 6A), which suggested that the former catalyst has higher generation/separation efficiencies for the photogenerated carriers than that of the latter catalyst (Huang et al., 2015). The electrochemical impedance spectra (EIS) was also conducted for two catalysts. It is accepted that the smaller radius of the arc, the smaller charge transfer resistance on the electrode surface, suggesting the faster transportation of the surface charge. As shown in Figure 6B, the data indicate that CMP-1 has a faster electron transfer process than that of CMP-2 because the semicircular radius of the CMP-1 is somewhat smaller than that of CMP-2 (Han et al., 2015). Photoluminescence spectroscopy was also conducted to compare the recombination rates of the electron/hole charge carriers of the catalysts. As shown in Figure 6C, the emission peak positions of the polymers locate at 470 nm for CMP-1 and 565 nm for CMP-2; the blue shift for CMP-1 was due to its wider band gap than that of CMP-2; and, then, the energy required the photon generation of CMP-1 is higher than that of the CMP-2 (Cui et al., 2020). The emission wavelength of CMP-1 is shorter than that of CMP-2, and the emission intensity of CMP-1 is significantly lower than that of CMP-2. The phenomenon reveals that CMP-1 has more sluggish recombination rate of the electron/hole charge carriers than that of CMP-2, which would make CMP-1 have more efficient HER than that of CMP-2 (Jayachandran and Chou, 2020). Time-resolved fluorescence spectroscopy (TRFS) was also conducted for evaluating the recombination rate of the electron/hole couples (Figure 6D), and the decay curves of the CMPs can be well fitted by a triple-exponential decay. The fluorescence lifetimes of CMP-1 were calculated to be τ_1_ = 0.7990 µs (78.34%) and τ_2_ = 6.6269 µs (21.66%), and the data for CMP-2 were calculated to be τ_1_ = 0.6284 µs (59.60%) and τ_2_ = 7.3962 µs (40.40%). The average lifetime of CMP-1 is 4.86 µs, which is shorter than that of CMP-2 (6.64 µs); this phenomenon indicates that CMP-1 has a higher separation efficiency for charge carriers (or excitons) than that of the CMP-2 (Gao et al., 2020). The fast separation efficiency for charge carriers is usually favorable to improve the photocatalytic activity of the catalysts.

### Photocatalytic H_2_ Production Evolution

**FIGURE 7:**
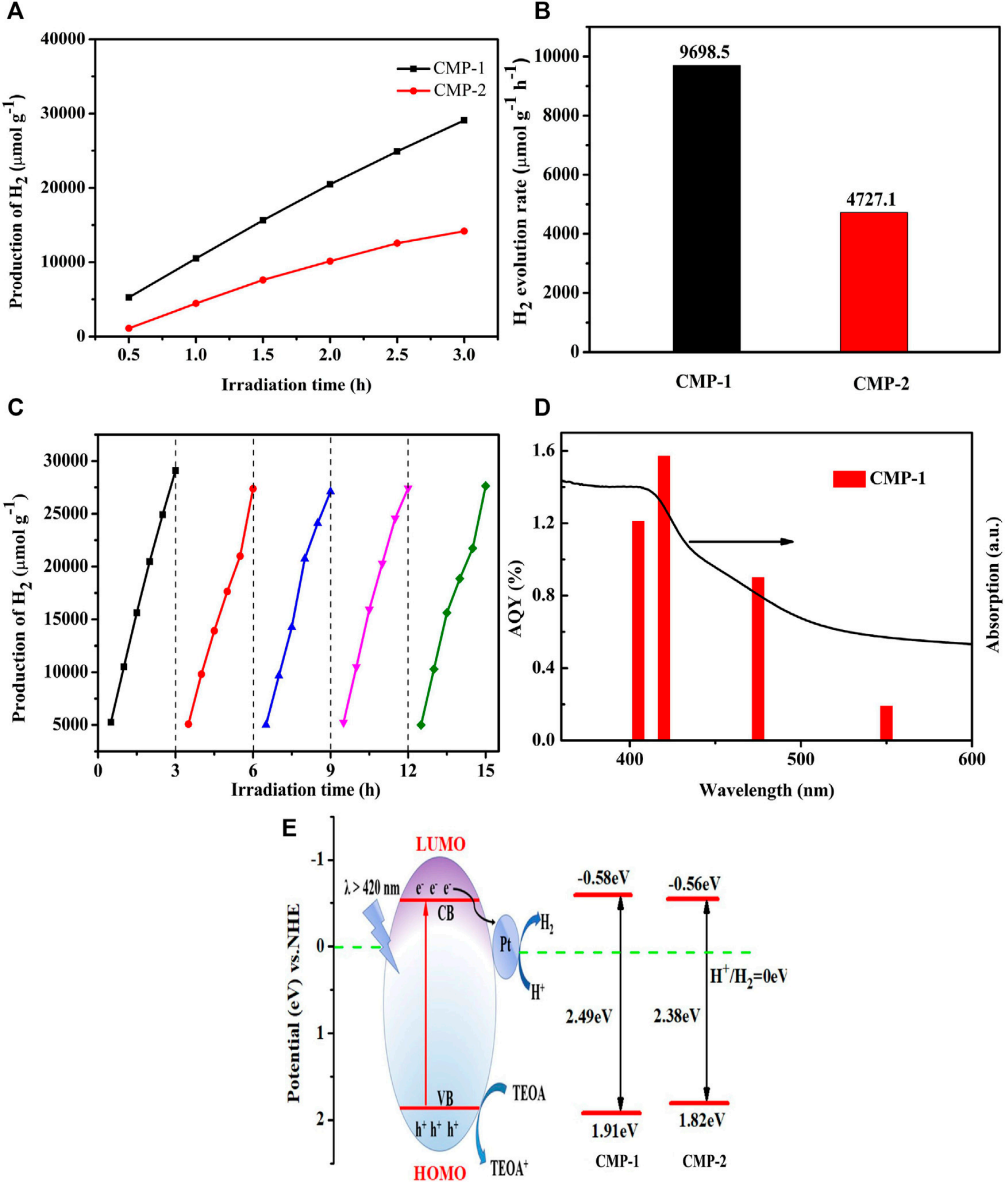
**(A)** The time-dependent hydrogen production of the CMP materials; **(B)** comparison of photocatalytic H_2_ evolution activity of CMP-1 and CMP-2; **(C)** the photocatalytic experiment with CMP-1 over five periods, 3 h for each period; **(D)** wavelength dependent AQY values of CMP-1, and a 300-W Xenon-lamp equipped with series of band-pass filters (405, 420, 455, and 550 nm) is used as light source; **(E)** energy diagram and photocatalytic hydrogen evolution mechanism.

The photocatalytic performance of the CMPs was measured using TEOA as the sacrificial agent under the illumination of visible light (≥420 nm), the HERs are recorded every 0.5 h for up to 3 h in five periods, and the resulting data are shown in Figure 7A. The average HER (Figure 7B) for CMP-1 is 9,698.5 μmol g^−1^ h^−1^ and the value for CMP-2 is about 4,727.1 μmol g^−1^ h^−1^; the HER for CMP-1 is twice as large as that of CMP-2. In addition; we also conducted the repeated irradiation experiments to study their stability as hydrogen evolution catalysts. As recorded in Figures 7A,C, slight decrease in HER was observed for CMP-1 at the second period. From then on and up to the fifth period, nearly no perceptible decline was observed, a reduction by 6% in HER occurs from the first period to the fifth period. The stability in HERs for CMP-2 (Supplementary Figure S8) was also measured for five periods and 3 h for each period. Compared with the first period, the HER for the fifth cycle decreased by 41%. This further indicates that the performance of CMP-1 is higher than that of CMP-2 in terms of HER and stability. Among the CMPs reported previously, the two CMPs in this study present fairly decent performance as shown in Table 1 (Ye et al., 2020; Yang et al., 2016; Cheng et al., 2018; Bi et al., 2017; Zhang et al., 2019). The apparent quantum yield (AQY) values of CMP-1 at four different wavelengths are given in Figure 7D, showing the consistence of its photocatalytic activity with its absorption curve. At the wavelength of 420 nm, the AQY value of CMP-1 is 1.57%, and CMP-1 catalyst can work under the broad absorption region between 360 and 600 nm with an AQY value of 0.19% at 550 nm. For comparison, the AQY values of CMP-2 are 0.81% and 0.11%, respectively, at 420 and 550 nm. Detailed control experiments were conducted to understand the indispensable characteristic of catalyst and light irradiation for hydrogen evolution process. In the process of hydrogen evolution experiment, the xenon lamp was turned off randomly, and the hydrogen production stopped increasing. When the light is turned on again, the hydrogen production returned to increasing. In addition, in the other control experiment, the experiment was conducted as the normal hydrogen production conditions except that the photocatalyst was not added in the Pyrex glass bottle, and, in this case, no hydrogen production was detected under the irradiation of the full spectrum light. After the purification process by Soxhlet extraction using chloroform, the residual Pd content of Pd was about 1,021 ppm for CMP-1 and 1,046 ppm for CMP-2. Although the residual Pd catalyst has been considered to play a significant role in the photocatalytic hydrogen production of conjugated polymers, even at ppm level concentrations, and in these studies, the additional Pt nanoparticles are not used as the co-catalyst (Kosco et al., 2018). With the addition of Pt co-catalyst, the residual Pd catalyst will play a very limited role on the hydrogen evolution activity of conjugated polymers.

**TABLE 1:** The photocatalytic H_2_ evolution rate of some CMPs.

Polymer	Optical gap (eV)	Light source^a^	HER (μmol h^−1^ g^−1^)	Cocatalyst	AQY^b^ (%)	References
B-BT-1,3,5	2.44	≥420 nm	400	Pt	-	Yang et al. (2016)
L-PyBT	2.23	≥420 nm	1,674	Pt	-	Cheng et al. (2018)
OB-POP-1	2.21	≥420 nm	134	Pt	-	Bi et al. (2017)
OB-POP-2	2.28	≥420 nm	598	Pt	-	Bi et al. (2017)
OB-POP-3	2.14	≥420 nm	908	Pt	2.0	Bi et al. (2017)
OB-POP-4	2.37	≥420 nm	620	Pt	-	Bi et al. (2017)
4-CzPN	2.11	≥420 nm	2,103.2	Pt	6.4	Zhang et al. (2019)
CMP-1	2.49	≥420 nm	9,698.5	Pt	1.57	[this work]
CMP-2	2.38	≥420 nm	4,727.1	Pt	0.11	[this work]

a Xe, Xenon lamp.

b At 420 nm; HER, hydrogen evolution rate; AQY, apparent quantum yield; TEOA, triethanolamine.

### Photocatalytic Mechanism

On the basis of the discussion, a hydrogen production mechanism (Figure 7E) over the present photocatalyst is proposed. The photocatalytic reaction is triggered as the light irradiation initiates the separation of the photogenerated carriers. The photoelectrons in the filled VB of the CMP are driven to the empty CB by the absorbed photons. The photogenerated electrons are captured by the FSO unit, which is considered as the electron-output “tentacle” and then transfers the electrons to the surface of the Pt co-catalyst (Dai et al., 2018). Electrons eventually combine with hydrogen protons in water on the surface of the Pt co-catalyst to produced hydrogen gas. The holes left in the VB are consumed by the oxidation reaction of TEOA (Wang Z. et al., 2018). It is found that CMP-1 has a faster electron/hole separation rate and a lower electron/hole recombination rate than that of CMP-2, although the difference between them only lies in that the benzene unit (as the arm unit) in CMP-1 is replaced by the thiophene unit in CMP-2. A higher LUMO level of CMP-1 allows the Pt Fermi energy to be raised to a higher level when electrons are transferred from the photocatalyst to Pt, thereby leading to a higher HER. Meanwhile, the HOMO of the CMP-1 has a slightly more positive potential than that of CMP-2, indicating that the generated holes in the VB of the CMP-1 have a greater oxidative capacity than that of the CMP-2 (Xiao et al., 2020). The above discussions explained the higher HER of CMP-1 than that of CMP-2.

## Conclusion

In summary, we reported here the design and synthesis of two CMPs containing benzene (or thiophene) armed triazine and FSO unit. The triazine and FSO-based CMPs offer now choices of robust photocatalysts with broad visible light absorption range and suitable energy level alignments. The benzene-containing CMP-1 has some advantages over the thiophene containing homolog CMP-2, including the lower recombination rate for electron/hole carriers and the higher separation and migration rates for the photogenerated charge carriers. As the bridging unit changes from benzene to thiophene, the HERs of the catalysts alter from 9,698.5 μmol g^−1^ h^−1^ to 4,727.1 μmol g^−1^ h^−1^. The difference in the catalytic performances obviously originates from the different linker unit employed. In addition, CMP-1 has higher molecular dipoles than that of CMP-2, which may cause a much faster charge mobility in CMP-1 than that in CMP-2. The present study demonstrates that the rational molecular design for CMP materials by selecting ideal bridging units is still one of the effective ways to obtain CMP-based photocatalysts toward hydrogen evolution from water splitting.

## Data Availability

The original contributions presented in the study are included in the article/Supplementary Material, further inquiries can be directed to the corresponding authors.
