# Supplementary material for: Triazine-Based Conjugated Microporous Polymers With Different Linkage Units for Visible Light–Driven Hydrogen Evolution
Source: Front Chem. 2022 Mar 25;10:854018. doi: 10.3389/fchem.2022.854018 (PMC8990882; doi:10.3389/fchem.2022.854018)
Supplement: Supplementary file 1 [file DataSheet1.docx]

Supporting Information

**Triazine based conjugated microporous polymers with different linkage units for visible light driven hydrogen evolution**

Qiannan Sheng^a^, Xiujuan Zhong^a+^, Qianqian Shang^a^, YunYun Dong^a^, Jinsheng Zhao^a,^*, Yuchang Du^b,^*, Yu Xie^c,^*

^a^College of Chemistry and Chemical Engineering, Liaocheng University, 252059, P.R. China.

^b^Key Laboratory of Jiangxi university for Applied Chemistry and Chemical Biology, College of Chemistry and Bioengineering, Yichun University, Yichun 336000, PR China

^c^College of Environment and Chemical Engineering, Nanchang Hangkong University, Nanchang 330063, PR China.

*Corresponding Author: [j.s.zhao@163.com](mailto:j.s.zhao@163.com) (J.S.Zhao); [yuchdu@126.com (Y.C. Du);](mailto:yuchdu@126.com;) [xieyu_121@163.com](mailto:xieyu_121@163.com)(Y. Xie)

^+^The same contribution

**Scheme. S1.** The structure of some Dibenzothiophene-S, S-dioxide (FSO) containing polymers including the conjugated porous polymers (P-FSO, S-CMP3 and PyDF) and the liner polymer FSO.

**Instruments and characterization methods**

X-ray photoelectron spectroscopy (XPS) measurements are conducted on a ESCALAB 250Xi spectrometer. FT-IR (Nicolet 6700) was used to get the spectrum of the polymers in the range of 500-4000 cm^-1^. Powder X-ray diffraction (PXRD) measurements are performed on a Rigaku D/max 2500 X-ray advance diffractometer, diffraction angles are selected from 5° to 80°. The morphology is characterized by Scanning Electron Microscope (SEM) (Thermo Fisher Scientific FIB-SEM GX4) and JEM-2100 transmission electron microscopy (TEM). The thermogravimetric analysis (TGA) of the materials is conducted on Netzsch STA449-C integrated thermal analyzer. The N_2_ adsorption-desorption isotherms are tested by Micromeritics ASAP 2460 analyzer at 77 K, and the samples are activated at 180 °C for 24 h prior to the test. UV-Vis-diffuse reflectance spectroscopy (UV-Vis-DRS) was performed using Cary 5000 UV-Vis-NIR spectrometer (Varian USA), with wavelength rang of from 200 nm to 1100 nm. The electrochemical measurements are conducted on a potentiostat (CHI 760D, CH Instruments) with a standard three-electrode system including the platinum plate electrode as the counter electrode, the Ag/AgCl as the reference electrode, and the catalyst modified glassy carbon electrode (GCE, Φ 3 mm) or the catalyst modified Indium-Tin Oxide (ITO) electrode as the working electrode. The catalyst slurry was prepared as follows: 10 mg of photocatalyst was dispersed into a mixture of 1 ml of isopropyl alcohol and 3 µL of naphthol, and be ultrasonicated for 30 min before use. Electrochemical impedance measurements (EIS) are conducted with amplitude of 50 mV and with frequencies ranging from 10^5^ to 0.1 Hz in 0.5 M Na_2_SO_4_ electrolyte, and the catalyst modified GCE electrode was used as the working electrode. The cyclic voltammetry (CV) of the polymers was conducted at a scan rate of 0.1 V/S with the catalyst modified GCE electrode in 0.1 M TBAPF_6_/ACN electrolyte. The Mott-Schottky tests were also performed with the catalyst modified GCE electrode in 0.5 M Na_2_SO_4_ as the supporting electrolyte. The catalyst modified GCE electrode was prepared by dipping 3 µL of the above polymer slurry on the GCE electrode, and be dried under room temperature. Photocurrent responses of the of the catalysts were measured using the joint utilization of the monochromator (CEL-SLF300) and the potentiostat (CHI 760D). A standard three-electrode system was used to control the potentials biased on the working electrode, the catalyst modified ITO electrode was used as the working electrode in 0.5 M Na_2_SO_4_ as the supporting electrolyte. The working electrode was made by dipping the catalyst slurry (20 µL) on the ITO electrode (1 cm × 1 cm), and be dried at room temperature before use. At the same time, a 300W xenon lamp with 420 nm filter was used as the light source. Photoluminescence (PL) and the time-resolved fluorescence spectroscopy (TRPL) were tested at fluorescence spectrometer (FLS1000), the excitation wavelength was set at 380 nm. The HOMO-LUMO energy levels were also examined using the DFT calculation with the B3LYP method with a basis set of 6-31*G. Electrostatic potential (ESP) maps of three polymer monomers were performed with the M062x functional and the def2TZVP basis set, using the Gaussian 16 program^[[1]](#endnote-1)^. The results were analyzed by Multiwfn.^^[[2]](#endnote-2)^^ A positive electrostatic potential represents electron-rich, a negative potential represents electron-poor.

The apparent quantum yield (AQY) of photocatalyst is also one of the factors for evaluating its photocatalytic activity. The AQY values of the CMPs are measured using the following equation (1):

| $AQY(\%)=\frac{2\times M\times N_{A}\times h\times c}{S\times P\times t\times\lambda}$ | (1) |
| --- | --- |

where M is the amount of H_2_ (mol), N_A_ is Avogadro constant (6.023×10^23^ mol^-1^), h is Planck constant (6.626×10^-34^ J/s), and c is speed of light in vacuum (3×10^8^ m/s), S is the illumination area (19.6 cm^2^), P is the incident light intensity (W/cm^2^), t is the irradiation time (s), and λ is the monochromatic wavelength (m). Monochromatic light was obtained by adding bandpass filters with different wavelengths on the xenon lamp light source. The light intensities of 405, 420, 455, 550 nm were 35.8, 37.2, 40.2, 50.4 mW/cm^2^ respectively, the illumination area was 19.6 cm^2^, and the illumination time was maintained at 1 h. To the pyrex glass reactor, 20 mg of photocatalyst, 10 mL of triethanolamine (TEOA, as sacrificial agent), 10 mL of NMP and 40 mL of distilled water were added. The catalyst was completely dispersed by ultrasound for 30 min, 24 µL of chloroplatinic acid was added, then the stirred solution was irradiated with a 300 W Xe lamp (CEL-HXF300) for 3 hours to load Pt nanoparticles (3%, w/w) on the the catalyst.

**Reagents and Materials**

Dibenzothiophene-5,5-dioxide, concentrated sulfuric acid, N-bromosuccinimide (NBS) bis(pinacolato)diboron, 1,1'-Bis(diphenylphosphino)ferrocene-palladium(II)dichloride dichloromethane complex(Pd(dppf)Cl_2_·DCM), N,N-dimethylformamide (DMF), Tetrabutylammonium hexafluorophosphate (TBAPF_6_), N-methypyrrolidone (NMP) are purchased from Shanghai Aladdin Biochemical Technology Co., Ltd. 2,4,6-tris(4-bromophenyl)-1,3,5-triazine (M2), 5-bromothiophene-2-carbonitrile and trifluoromethanesulfonic acid are obtained from Zhengzhou alpha Chemical Co. Ltd. Chloroform, methanol, ethanol, acetone, triethanolamine, N-hexane, dichloromethane, acetonitrile (ACN) are obtained from Tianjin Kemio Chemical Reagent Co., Ltd. 3,7-bis(4,4,5,5-tetramethyl-1,3,2-dioxaborolan-2-yl)dibenzo[b,d] thiophene 5,5-dioxide (M1), 3,7-dibromodibenzothiophene-*S,S*-dioxide was prepared according to the method in previous report ^[1]^. 2,4,6-tris(4-bromophenyl)-1,3,5-triazine (M2) was obtained from Zhengzhou alpha Chemical Co. Ltd. 2,4,6-Tris(5-bromothiophene-2-yl)-1,3,5-triazine (M3) was synthesized according to the previous report ^[2]^.

**1. Synthesis of 3,7-dibromodibenzothiophene-S, S-dioxide**

NBS (1.64 g, 9,24 mmol) was added into this solution of dibenzothiophene-S, S-dioxide (1 g, 4.62 mmol) in concentrated H_2_SO_4_ (30 mL) in several portions, and the resulting mixture was stirred at 0 °C for 24 h. The mixture was carefully poured into ice/water. The off-white solid was filtered off, washed with 20% aqueous sodium hydrogen carbonate, water and dried to afford white solid. The product was further recrystallized from chloroform to gain white crystal in 60% yield. ^1^H-NMR (400 MHz, CDCl_3_) δ 7.93 (s, 2H), 7.78 (d, 2H), 7.65 (d, 2H). ^13^CNMR (400 MHz, CDCl_3_), δ, 138.96, 137.25, 129.68, 125.70, 123.02.

**2. Synthesis of 3,7-bis(4,4,5,5-tetramethyl-1,3,2-dioxaborolan-2-yl)dibenzo[b,d] thiophene 5,5-dioxide (M1)**

Under nitrogen, a two-necked flask (100 mL) was charged with 3,8-dibromodibenzothiophene-S, S-dioxide (1.01 g, 2.70 mmol), bis(pinacolato)diboron (1.49 g, 5.88 mmol), anhydrous KOAc (1.57 g, 15.99 mmol), dppf (89 mg, 0.16 mmol) and dry dioxane (50 mL) and degassed with argon for 15 min. Pd(dppf)Cl_2_·DCM (131 mg, 0.16 mmol) was added and the mixture was degassed for another 15 min. The mixture was heated at 100 ºC for 32 hours under argon atmosphere. After cooling to room temperature, the solvent was removed under reduced pressure and the residue was diluted with water (50 mL). The aqueous solution was extracted with DCM (2×50 mL), washed with water (2 ×20 mL) and dried with anhydrous MgSO_4_. The DCM solution (dark color) was then passed through a short silica gel bed eluting with dichloromethane (100 mL) to give a clear solution which was concentrated to afford the products 8 as an off white solid (1.01 g, 85%). ^1^H NMR (400 MHz, CDCl_3_): δ (ppm) 8.28 (2H, s), 8.05 (2H, d, J=7.7 Hz), 7.81 (2H, d, J= 7.9 Hz), 1.36 (24H, s). ^13^C NMR (101 MHz, CDCl_3_): δ (ppm): 140.05 (CH), 137.50(CH), 133.73(CH), 132.37(br.), 128.33(CH), 121.09 (CH), 84.56, 24.87 (CH_3_).


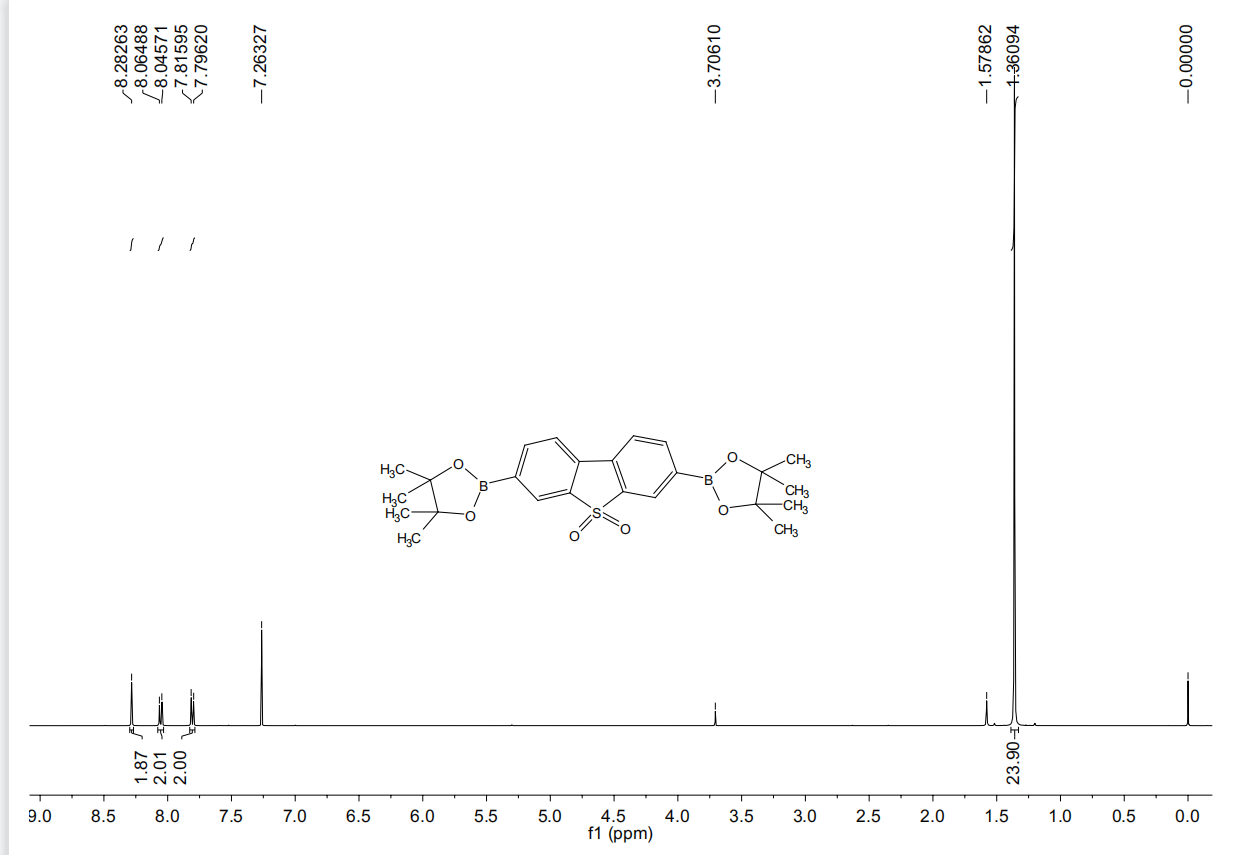


**Figure. S1**. ^1^H NMR spectrum of M1.

2,4,6-tris(4-bromophenyl)-1,3,5-triazine (M2) was obtained from Zhengzhou alpha Chemical Co. Ltd. The structure of the molecule was as follows:

**3. Synthesis of 2,4,6-Tris(5-bromothiophene-2-yl)-1,3,5-triazine (M3)**^[2]^

4.0 g (21.3 mmol) of 5-bromothiophene-2-carbonitrile was dissolved in 500 mL of dry chloroform, and then 12.8 g of (85.2 mmol) of trifluoromethanesulfonic acid was dropped into the solution at at 0 °C. The resultant solution was magnetically stirred for another 2 hours at 0 °C, and then, the temperature of the solution was risen to room temperature for 48 h. The mixture was rinsed with distilled water, and dried by anhydrous magnesium sulphate. The solution was obtained by filtration. And then the solvent was distilled off by vacuum distillation. The solid as crude product was purified with recrystallization in toluene, affording the product as white needle. ^1^H NMR (500 MHz, Chloroform-*d*), δ, 7.97 (d, *J* = 4.0 Hz, 3H), 7.17 (d, *J* = 4.0 Hz, 3H). ^13^C NMR (126 MHz, CDCl_3_), δ, 142.40, 136.54, 134.56, 130.10, 129.47, 127.46, 127.35, 126.82, 124.74.








**Figure. S2**. (a) ^1^H NMR spectrum of M3, (b) ^13^C NMR spectrum of M3.

[1] C. Shu, C.Z. Han, X.Y. Yang, C. Zhang, Y. Chen, S.J. Ren, F. Wang, F. Huang, J.S. Jiang. Boosting the photocatalytic hydrogen evolution activity for D-π-A conjugated microporous polymers by statistical copolymerization. *Adv. Mater.* ***2021****, 33, 2008498*.

[2] X. Xue, J.M. Luo, L.Q. Kong, J.S. Zhao, Y. Zhang, H.M. Du, S. Chen, Y. Xie. The synthesis of triazine-thiophene-thiophene conjugated porous polymers and their composites with carbon as anode materials in lithium-ion batteries. *RSC Advance*. ***2021****,11, 10688-10698.*


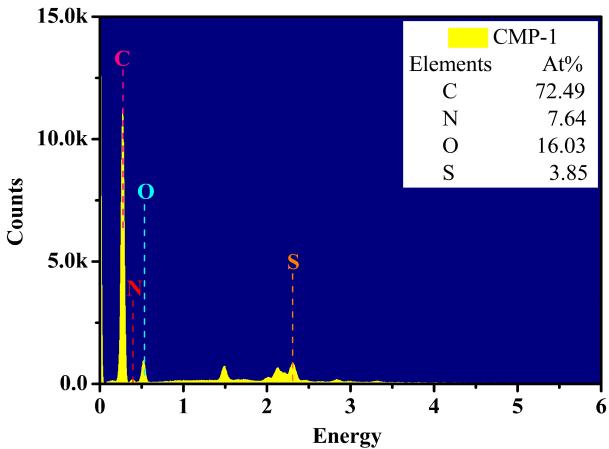

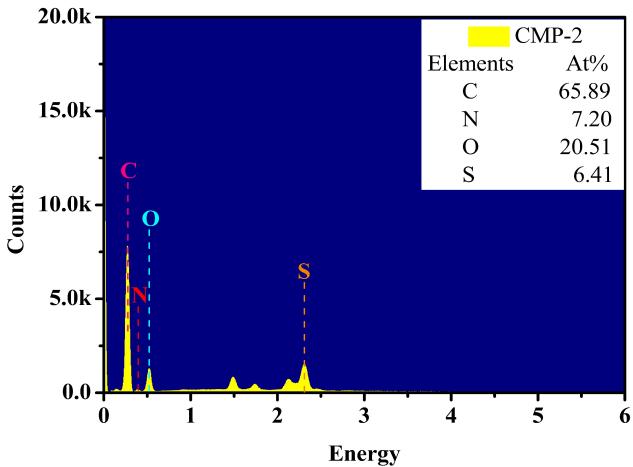


**Figure. S3.** EDX measurements of CMP-1, CMP-2.


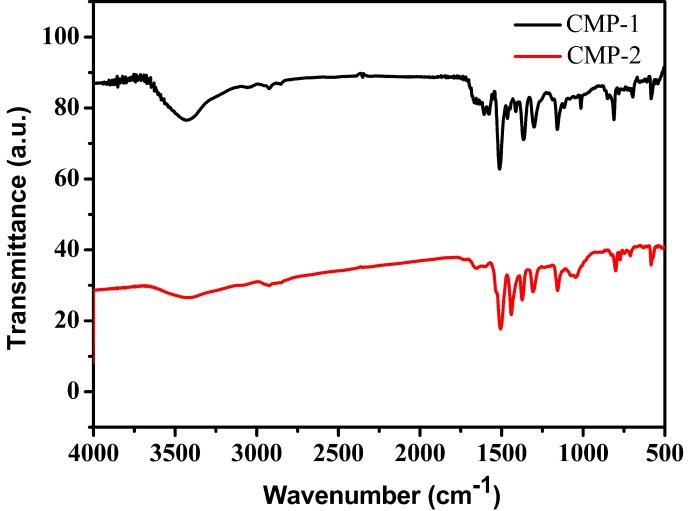


**Figure. S4.** FT-IR of CMPs after its reaction.


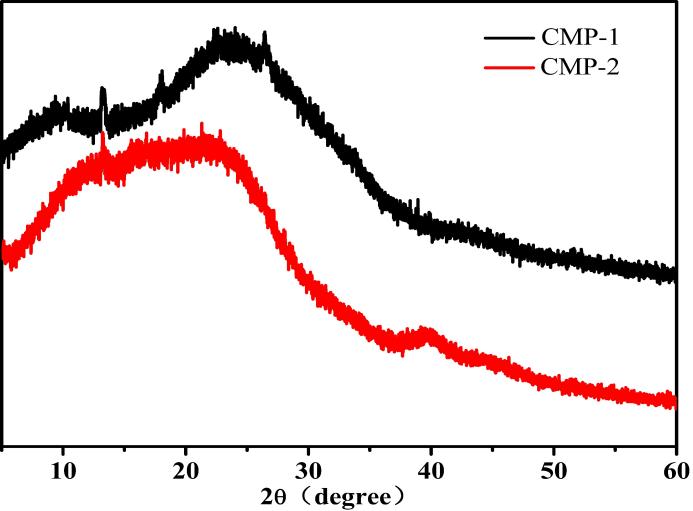


**Figure. S5.** XRD of the two CMP catalysts.


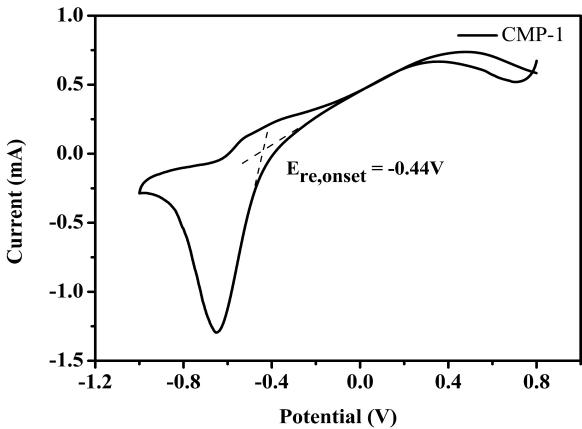

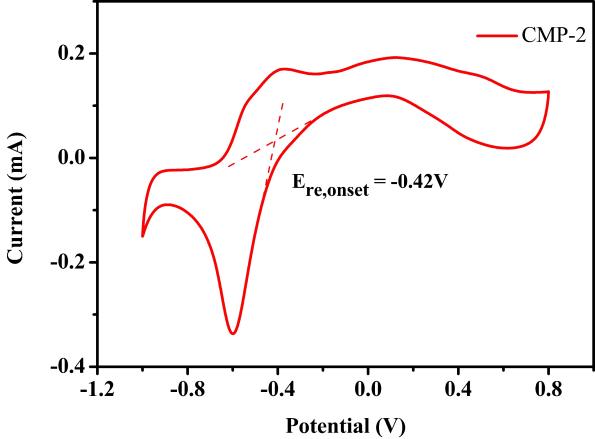


**Figure. S6.** Cyclic voltammetry measurements of CMP-1 and CMP-2 (in 0.1M MeCN-NBu_4_PF_6_)


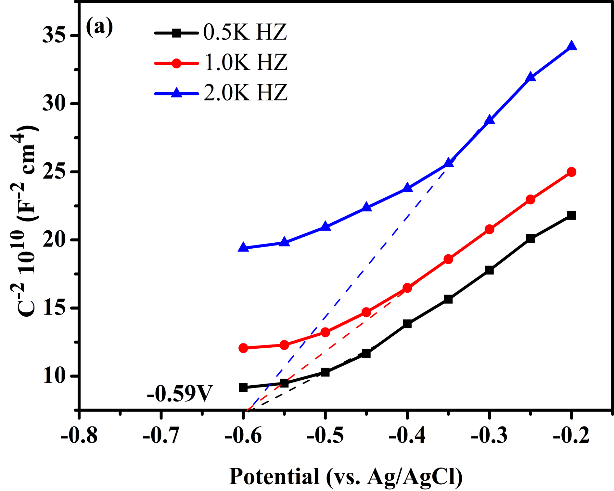

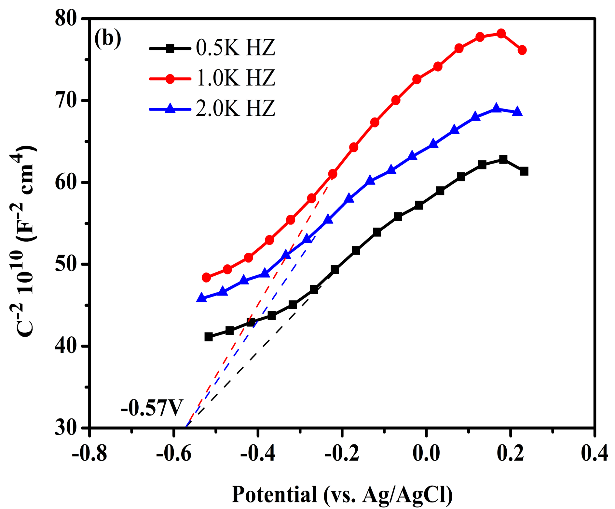


**Figure. S7.** Mott-Schottky plots spectra for CMP-1and CMP-2.


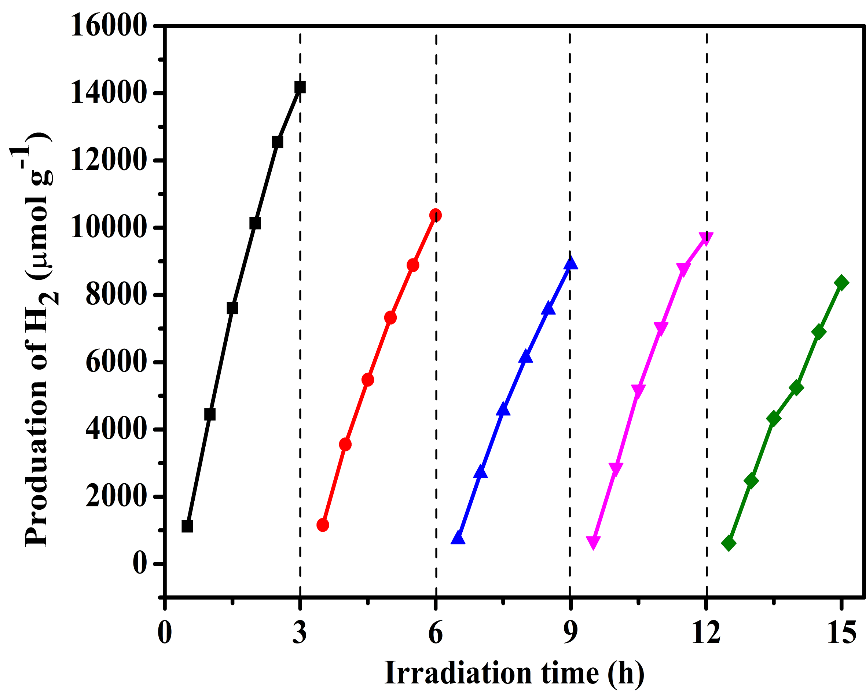


**Figure. S8.** Long-time photocatalytic experiment with CMP-2 over five periods of photocatalytic reaction, 3 hours for each period.

1. Gaussian 16, Revision C.01, M. J. Frisch, G. W. Trucks, H. B. Schlegel, G. E. Scuseria, M. A. Robb, J. R. Cheeseman, G. Scalmani, V. Barone, G. A. Petersson, H. Nakatsuji, X. Li, M. Caricato, A. V. Marenich, J. Bloino, B. G. Janesko, R. Gomperts, B. Mennucci, H. P. Hratchian, J. V. Ortiz, A. F. Izmaylov, J. L. Sonnenberg, D. Williams-Young, F. Ding, F. Lipparini, F. Egidi, J. Goings, B. Peng, A. Petrone, T. Henderson, D. Ranasinghe, V. G. Zakrzewski, J. Gao, N. Rega, G. Zheng, W. Liang, M. Hada, M. Ehara, K. Toyota, R. Fukuda, J. Hasegawa, M. Ishida, T. Nakajima, Y. Honda, O. Kitao, H. Nakai, T. Vreven, K. Throssell, J. A. Montgomery, Jr., J. E. Peralta, F. Ogliaro, M. J. Bearpark, J. J. Heyd, E. N. Brothers, K. N. Kudin, V. N. Staroverov, T. A. Keith, R. Kobayashi, J. Normand, K. Raghavachari, A. P. Rendell, J. C. Burant, S. S. Iyengar, J. Tomasi, M. Cossi, J. M. Millam, M. Klene, C. Adamo, R. Cammi, J. W. Ochterski, R. L. Martin, K. Morokuma, O. Farkas, J. B. Foresman, and D. J. Fox, Gaussian, Inc., Wallingford CT, 2016. [↑](#endnote-ref-1)
2. T. Lu and F. W. Chen, *J. Comput. Chem.*, 2012, 33, 580−592. [↑](#endnote-ref-2)
